# Exploiting the Role of Endogenous Lymphoid-Resident Dendritic Cells in the Priming of NKT Cells and CD8+ T Cells to Dendritic Cell-Based Vaccines

**DOI:** 10.1371/journal.pone.0017657

**Published:** 2011-03-31

**Authors:** Troels R. Petersen, Dianne Sika-Paotonu, Deborah A. Knight, Helen M. A. Simkins, Ian F. Hermans

**Affiliations:** 1 Malaghan Institute of Medical Research, Wellington, New Zealand; 2 School of Biological Sciences, Victoria University of Wellington, Wellington, New Zealand; Southern Illinois University School of Medicine, United States of America

## Abstract

Transfer of antigen between antigen-presenting cells (APCs) is potentially a physiologically relevant mechanism to spread antigen to cells with specialized stimulatory functions. Here we show that specific CD8^+^ T cell responses induced in response to intravenous administration of antigen-loaded bone marrow-derived dendritic cells (BM-DCs), were ablated in mice selectively depleted of endogenous lymphoid-resident langerin^+^ CD8α^+^ dendritic cells (DCs), suggesting that the antigen is transferred from the injected cells to resident APCs. In contrast, antigen-specific CD4^+^ T cells were primed predominantly by the injected BM-DCs, with only very weak contribution of resident APCs. Crucially, resident langerin^+^ CD8α^+^ DCs only contributed to the priming of CD8^+^ T cells in the presence of maturation stimuli such as intravenous injection of TLR ligands, or by loading the BM-DCs with the glycolipid α-galactosylceramide (α-GalCer) to recruit the adjuvant activity of activated invariant natural killer-like T (*i*NKT) cells. In fact, injection of α-GalCer-loaded CD1d^−/−^ BM-DCs resulted in potent *i*NKT cell activation, suggesting that this glycolipid antigen can also be transferred to resident CD1d^+^ APCs. While *i*NKT cell activation *per se* was independent of langerin^+^ CD8α^+^ DCs, some *i*NKT cell-mediated activities were reduced, notably release of IL-12p70 and transactivation of NK cells. We conclude that both protein and glycolipid antigens can be exchanged between distinct DC species. These data suggest that the efficacy of DC-based vaccination strategies may be improved by the incorporation of a systemic maturation signal aimed to engage resident APCs in CD8^+^ T cell priming, and α-GalCer may be particularly well suited to this purpose.

## Introduction

Migratory DCs perform a sentinel role as immature cells in the peripheral tissues. They respond to local danger signals, such as factors released by perturbed tissue in response to infection, by migrating to the draining lymphoid tissues with antigens they have acquired from the local environment. In the process migratory DCs undergo maturation, characterized by increased expression of costimulatory molecules, adhesion molecules and MHC molecules, to provide a stimulatory phenotype capable of driving activation and proliferation of antigen-specific T cells. There is also a level of constitutive migration of DCs from peripheral tissues to the lymphoid organs in the absence of danger, so that sizable populations of DCs with migratory phenotype can be detected in lymph nodes and spleen [1]. So-called ‘lymphoid-resident’ DCs (referred to as resident DCs), on the other hand, seed the lymphoid tissues directly as precursors released from the bone marrow. In the steady state, lymphoid-resident DCs represent approximately half of the DCs in the lymph nodes, and the majority of DCs in the spleen [1], [2]. In mouse spleen three populations of lymphoid-resident DC can be distinguished on the basis of expression of CD4 or CD8α (CD4^+^ DC, CD8α^+^ DC, or double negative DC) [2], [3], [4], with some functional specialization between the subsets. In particular, CD8α^+^ DCs have a heightened capacity to cross-present soluble and cell-associated antigens via MHC class I molecules to CD8^+^ T cells [5], [6], [7], [8], and are major producers of IL-12 in response to microbial activation stimuli [9], [10].

Lymphoid-resident DCs have been generally assumed to acquire antigens and danger signals directly from blood or lymph circulating through the lymphoid tissues. A less appreciated mechanism of acquiring antigen involves transfer from migratory DCs to lymphoid-resident DCs. It has been shown that migratory DCs that drain the skin following herpes simplex virus infection can transfer antigens to lymph node-resident CD8α^+^ DCs, which are then responsible for stimulating CD8^+^ T cell responses [11]. A similar transfer process between migrating DCs and resident CD8α^+^ DCs has been reported in lymph-nodes draining the lung following influenza infection [12], suggesting antigen transfer is a physiologically relevant mechanism for spreading antigen to specialized antigen-presenting cells to enhance specific T cell responses. The phenotype and maturation status of the recipient lymphoid-resident DC populations may therefore be a critical factor in driving effective immune responses. In the case of an infection such as herpes simplex virus, it is possible that infection-driven maturation of recipient DCs ultimately drives the T cell response. In other situations, effective immunity may be reliant on circulation of factors capable of triggering maturation of lymphoid-resident DCs. In the absence of these stimuli, regardless of the maturation state of the migratory DC, the resident DCs may fail to stimulate immunity, or could potentially alter the response through, for example, the induction of T regulatory cells [13]. This possibility may be particularly relevant in the context of DC vaccination, as transfer of antigen from injected DCs to endogenous antigen presenting cells has been reported in animal models [14], [15], [16], and has been suggested to contribute significantly to the vaccine induced CD8^+^ T cell responses [17]. Attempts to induce anti-tumor T cell responses in cancer patients typically involve culture of autologous DCs with tumor tissue or specific tumor antigens, followed by a DC maturation step prior to injection. While this maturation step may enhance migration of the injected DCs from the site of injection to the draining LN, and enhance T cell priming by ensuring adequate expression of costimulatory markers, it may not support effective T cell stimulation by resident DCs that acquire antigen from injected cells.

We reasoned that CD8^+^ T cell activation in response to DC vaccination could be enhanced by strategies aimed at optimizing the function of lymphoid-resident DC capable of cross-presentation. In the mouse, a subset of lymphoid-resident CD8α^+^ DC expressing the c-type lectin langerin (CD207) has been shown to be primarily responsible for cross-presentation and IL-12 production following injection of soluble antigen and adjuvant [18]. We therefore investigated the role of langerin^+^ CD8α^+^ DCs after injection of antigen-loaded DCs in a transgenic animal model in which langerin^+^ cells can be specifically depleted in vivo. We show that langerin^+^ CD8α^+^ can indeed capture antigen from intravenously injected DCs, and can significantly contribute to induced CTL responses, but only if they receive appropriate maturation stimuli. In this context, loading the injected DCs with α-galactosylceramide (α-GalCer) to provoke ‘licensing’ by *i*NKT cells [19], [20], [21], [22], [23], [24] proved to be particularly effective, as this glycolipid antigen was also effectively transferred to resident DCs, allowing the recipient cells to also become licensed for CTL induction. Thus, transfer of protein and glycolipid antigens to lymphoid resident DCs is a feature of DC vaccination that can be exploited to improve vaccine outcome.

## Materials and Methods

### Mice

Breeding pairs of the inbred strains C57BL/6j (H2^b^, CD45.2^+^) and B6.Sjptprca (H2^b^, CD45.1^+^) were obtained from the Animal Resource Centre, Canning Vale, Western Australia. Also used were B10/Q-H2^q^/SgAi mice (H2^q^) (referred to as B10.Q), (Taconic Repository, NIH, Bethesda MD, USA), OT-I mice expressing a transgenic Vα2, Vβ5.1/5.2 T cell receptor (TCR) specific for an H-2K^b^-binding peptide of chicken ovalbumin (OVA_257–264_) [25], OT-II mice, expressing a transgenic Vα2, Vβ5.1/5.2 TCR recognizing the I-A^b^-restricted epitope OVA_323–339_ [26], Langerin-DTREGFP (referred to as *lang*-DTREGFP) mice expressing the human *Diphtheria* toxin (DT) receptor under control of the langerin promoter [27] (kindly provided by B. Malissen, Marseille, France), CD1d^−/−^ mice [28] (kindly provided by C-R Wang, University of Chicago, Illinois, USA), which are devoid of Vα14 *i*NKT cells, and TLR-4^−/−^ mice [29] (kindly provided by Dr S Akira, Hyogo College of Medicine, Japan). For some adoptive transfer experiments OT-I or OT-II animals were crossed with B6.Sjptprca animals (referred to as CD45.1/OT-I and CD45.1/OT-II), so that the congenic marker CD45.1 could be used to discriminate the transferred cells. All mice were maintained by the Biomedical Research Unit, Malaghan Institute of Medical Research, Wellington, New Zealand. Experimental protocols were approved by Victoria University Animal Ethics Committee (permit number 2007R17M), and performed according to their guidelines. Mice used in this study were 7-10 weeks of age and, when necessary, matched for age and gender.

### Culture media and reagents

Bone marrow-derived DCs (BM-DCs) were cultured in complete IMDM (cIMDM) consisting of IMDM medium (Invitrogen, Auckland, New Zealand) supplemented with 5% FBS (Sigma-Aldrich, Auckland, New Zealand), and 2 mM glutamax, 100 U/ml penicillin, 100 µg/ml streptomycin, and 50 µM 2-mercaptoethanol (all Invitrogen). The *i*NKT cell ligand α-GalCer was manufactured as described in [30] and solubilized in 150 mM NaCl, 0.5% Tween 20. Endotoxin free chicken OVA was purchased from Profos AG (Profos AG, Regensburg, Germany). The toll-like receptor (TLR) ligands used were polyinosinic polycytidylic acid (p(I:C)) (InvivoGen, San Diego, CA, USA) and monophosphoryl lipid A (MPL) (Sigma-Aldrich). The antibodies used were: anti-Vα2 (clone B20.1), anti-IFN-γ (clone XMG1.2), anti-B220 (clone RA3-6B2), anti-TCR-β (clone H57-597), and anti-Vβ5.1/5.2 (clone MR9-4) from BD-Pharmingen (BD-Pharmingen, Auckland, New Zealand), and anti-IL-4 (clone 11B11), anti-CD4 (clone 11B11), anti-CD8α (clone 53–6.7), anti-CD86 (clone GL1), and anti-CD45.1 (clone A20) from eBioscience (eBioscience, San Diego, CA, USA). Propidium iodide (PI) was from Sigma, CFSE from Molecular Probes (Molecular Probes, Invitrogen, New Zealand). Diphtheria toxin (DT) was from Sigma-Aldrich.

### Generation, antigen loading, and injection of bone marrow-derived DCs

Bone marrow-derived dendritic cells (BM-DCs) were generated by culturing bone-marrow cells from the femur and tibia of 7–10 week old mice in cIMDM media with 10 ng/ml GM-CSF and 20 ng/ml IL-4 in 5 ml of complete medium, with half of the medium replaced by new medium containing GM-CSF and IL-4 on day 2 and 5. On day 6, cultures were added OVA at 1 mg/ml and/or α-GalCer at 200 ng/ml. In some cultures OVA was added with MPL at 100 ng/ml. After overnight incubation, loosely attached cells were spun down and washed at least three times in IMDM, and 5×10^5^ DCs were injected intravenously in 200 µl IMDM. In some experiments, 25 µg MPL, 100 µg p(I:C), or 200 ng α-GalCer was injected *i.v.* in the contralateral tail vein after administration of OVA-loaded BM-DCs.

### Flow cytometry

All antibody labeling was performed on ice in FACS buffer (PBS supplemented with 1% FCS, 0.05% sodium azide, and 2 mM EDTA). Non-specific FcR-mediated antibody staining was blocked by incubation for 10 min with anti-CD16/32 Ab (24G2, prepared in-house from hybridoma supernatant). Flow cytometry was performed on a BD Biosciences FACSCalibur or BD LSRII SORP flow cytometer with data analysis using FlowJo software (Tree Star, Inc., OR, USA).

### Tracking of T cell responses

To facilitate detection of OVA-specific T cells, mice received a cohort of 5×10^4^ LN cells from CD45.1/OT-I mice or 5×10^6^ LN cells from CD45.1/OT-II mice. Mice were tail-bled 7 days after vaccination and the percentage of OT-I or OT-II cells of all CD8^+^ or CD4^+^ cells respectively, was determined by flow cytometry. In some experiments, OT-II cells were labeled with 1 µM CFSE before injection, and the OT-II T cell proliferation was assessed in the spleen three days after DC vaccination.

### *In vivo* depletion of langerin^+^ cells

*Lang*-DTREGFP mice were injected *i.p.* with 350 ng of DT 48 h and 24 h day prior to administration of DCs to deplete langerin^+^ cells [18]. In some experiments, control C57BL/6j mice were similarly injected with DT.

### Pentamer staining and *in vivo* killing assay

*Lang*-DTREGFP mice were injected with DT or PBS and vaccinated with C57BL/6j BM-DCs loaded with OVA and α-GalCer as described above. The percentage of CD8^+^ T cells specific for the OVA_257–264_-peptide was determined in the blood 6 days later by flow cytometry using anti-CD8 antibody and fluorescently labeled K^b^/OVA_257–264_ pentamers (ProImmune, Oxford, UK) according to manufacturers instructions. The following day, in vivo cytotoxicity was assessed against syngeneic spleen cell populations loaded with OVA_257–264_ peptide and administered by i.v. injection. Mixtures of three populations were injected: a control population without peptide fluorescence-labeled with 10 µM of the dye chloromethyl-benzoyl-aminotetramethyl-rhodamine (CTO) and two target populations loaded with 50 or 5 nM OVA_257–264_ peptide and fluorescence-labeled with CFSE at different dye concentrations (1.65 and 0.3 nM respectively). Peptide-specific lysis was assessed by flow cytometry of blood 16 h later, with the mean percent survival of peptide-loaded targets cells calculated relative to that of peptide-negative controls. Cytotoxic activity was expressed as the percent specific lysis, calculated by the equation 100 - mean percent survival of peptide-loaded targets.

### Intracellular cytokine staining of *i*NKT cells

Mice were injected *i.v.* with 5×10^5^ α-GalCer-loaded BM-DCs. The mice were sacrificed 2 h later, and single cell suspensions prepared from spleens were depleted of red blood cells (RBC lysis buffer, Qiagen), washed twice with cold FACS buffer, and incubated with anti-Fc receptor antibody 2.4G2 to block non-specific antibody binding. The cells were then incubated with CD1d/α-GalCer tetramer (ProImmune) for 20 min on ice followed by incubation with anti-B220 and anti-TCR-β antibodies for 15 min before being fixed overnight at 4°C in 4% formaldehyde (Sigma-Aldrich). The cells were then washed twice in saponin buffer (PBS supplemented with 0.1% BSA, 0.1% saponin, and 0.05% NaN_3_ (all Sigma-Aldrich), before incubation with anti-IL-4, anti-IFN-γ, or isotype control antibody, in saponin buffer on ice for 15 min. The cells were finally washed twice in saponin buffer, resuspended in FACS buffer, and analyzed on a BD FacsCalibur. For analysis, B220^+^ cells were excluded, and *i*NKT cells gated as TCR-β^+^ CD1d/α-GalCer^+^ cells.

### Analysis of cytokine release into serum

Blood was collected from the lateral tail vein at different time intervals after BM-DC administration. Serum was collected after blood had clotted, and levels of the cytokines IL-12p70, IL-4, and IFN-γ were assessed by bioplex cytokine bead arrays (Bio-Rad) according to the manufacturer's instructions.

### Uptake of BM-DCs by spleen DCs

BM-DCs generated from B6.Sjptprca (H2^b^, CD45.1^+^) mice were labeled with 1.5 µM CFSE, and 10×10^6^ BM-DCs were injected *i.v.* in C57BL/6j mice. Spleens were removed 9–16 h later, and digested with liberase and DNAse I to aid release of resident antigen-presenting cells (both Roche, Auckland, New Zealand). DCs were isolated from the splenocyte preparations with magnetic sorting (CD11c-MACS MicroBeads, clone N418; Miltenyi Biotec, Bergisch Gladbach, Germany) according to manufacturers instructions, and analyzed by flow cytometry with antibodies against CD11c, CD8α and CD45.2, and using DAPI (Invitrogen) to discriminate viable cells.

### Statistical analyses

A Mann-Whitney test was used to determine statistical significance in experiments with two experimental groups. In experiments with three or more groups, statistical significance was determined using a Krusskal-Wallis test with Dunn's post test used to determine statistical significance between two individual groups. All statistical analyses were done with GraphPad Prism software (GraphPad Software, Inc., La Jolla, CA, USA).

## Results

### Resident APCs cross-present antigen acquired from injected BM-DCs

**Figure 1:**
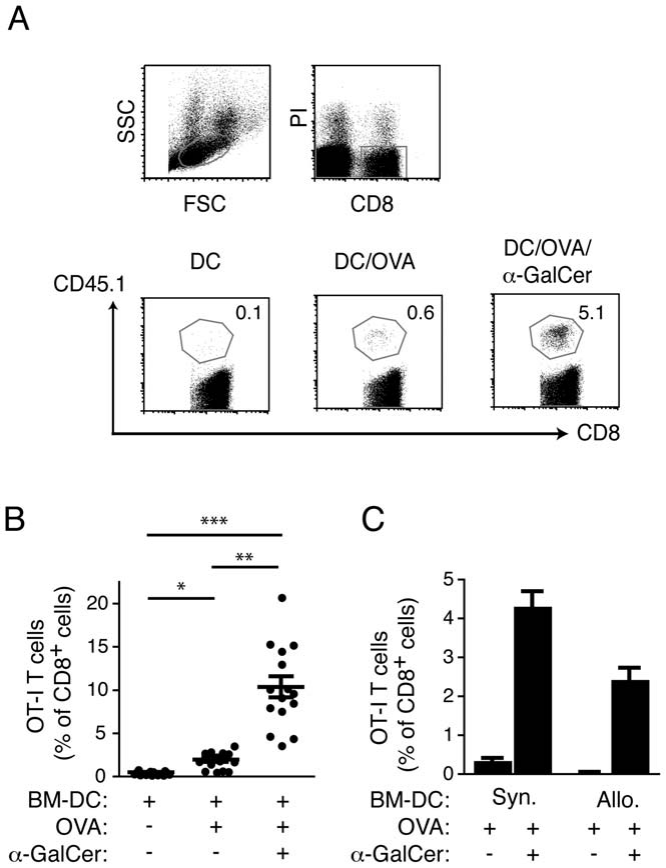
Antigen from injected allogeneic BM-DCs is cross-presented by resident APCs. C57BL/6j mice (CD45.2) were provided a cohort of OVA-specific CD8^+^ T cells from OT-I mice (CD45.1) by adoptive transfer and were vaccinated 1 d later with BM-DCs derived from syngeneic mice that had been loaded with OVA ± α-GalCer *in vitro*. Accumulation of OT-I cells was determined in blood 7 d later by flow cytometry with antibodies specific for CD8 and CD45.1. (*A*) Flow cytometry data outlining the gating strategy for the identification of OT-I T cells, and representative dot plots showing OT-I T cells as a percentage of all CD8^+^ cells in blood on day 7. (*B*) Pooled data ± SEM from three experiments with 4–5 mice per group are shown with each symbol representing an individual mouse. Statistical significance was determined using a Kruskal-Wallis test and Dunn's post test. *p<0.05, **p<0.01, ***p<0.001 (*C*) Mice were vaccinated with either syngeneic (C57BL/6j) or allogeneic (B10.Q) BM-DCs loaded with OVA ± α-GalCer and the percentage of OT-I T cells of all CD8^+^ cells determined as above. One representative experiment out of two with 4-5 mice per group is depicted with SEM.

Vaccination with protein-loaded DCs has been demonstrated to prime antigen-specific CD4^+^ and CD8^+^ T cell responses, but the contribution of resident DCs in this process remains unclear. To establish whether resident DCs acquire and present antigen derived from injected DCs, we examined the *in vivo* priming of a cohort of OVA-specific CD8^+^ transgenic T cells (OT-I T cells) in C57BL/6j recipients injected with OVA-loaded BM-DCs from syngeneic (C57BL/6j; H-2^b^) or allogeneic (B10.Q; H-2^q^) animals. As OT-I T cells recognize OVA as a processed peptide (OVA_257–264_) presented by H-2K^b^ molecules, only syngeneic BM-DCs can provide a direct stimulus to these T cells; any OT-I T responses initiated after injection of allogeneic H-2^q^ BM-DCs must therefore involve “indirect” cross-presentation via resident APCs. For these experiments, the BM-DCs were also loaded with the *i*NKT-cell ligand α-GalCer, which significantly enhances T cell responses by promoting *i*NKT cell-mediated licensing of APCs [19], [20], [21], [22], [23], [24]. To ensure interaction with *i*NKT cells, the BM-DCs were administered *i.v*., thereby gaining access to the *i*NKT cell-rich areas of the spleen. The accumulation of OT-I T cells was determined in the blood 7 days after BM-DC administration (Fig. 1*A*). As expected, injection of syngeneic BM-DCs loaded with OVA alone induced a moderate expansion of the OT-I T cell cohort, while BM-DCs loaded with both OVA and α-GalCer provoked a much stronger response due to the adjuvant activity of stimulating *i*NKT cells (Fig. 1*B*). Interestingly, injection of allogeneic BM-DCs loaded with OVA and α-GalCer also resulted in accumulation of OT-I T cells, although to lower levels than syngeneic BM-DCs (Fig. 1*C*), despite the fact that the allogeneic BM-DCs were unable to directly stimulate the transgenic T cells. We conclude that antigen can be transferred from injected DCs to resident APCs for processing and indirect cross-presentation to CD8^+^ T cells.

### Depletion of langerin^+^ DCs ablates indirect antigen-presentation by resident DCs

**Figure 2:**
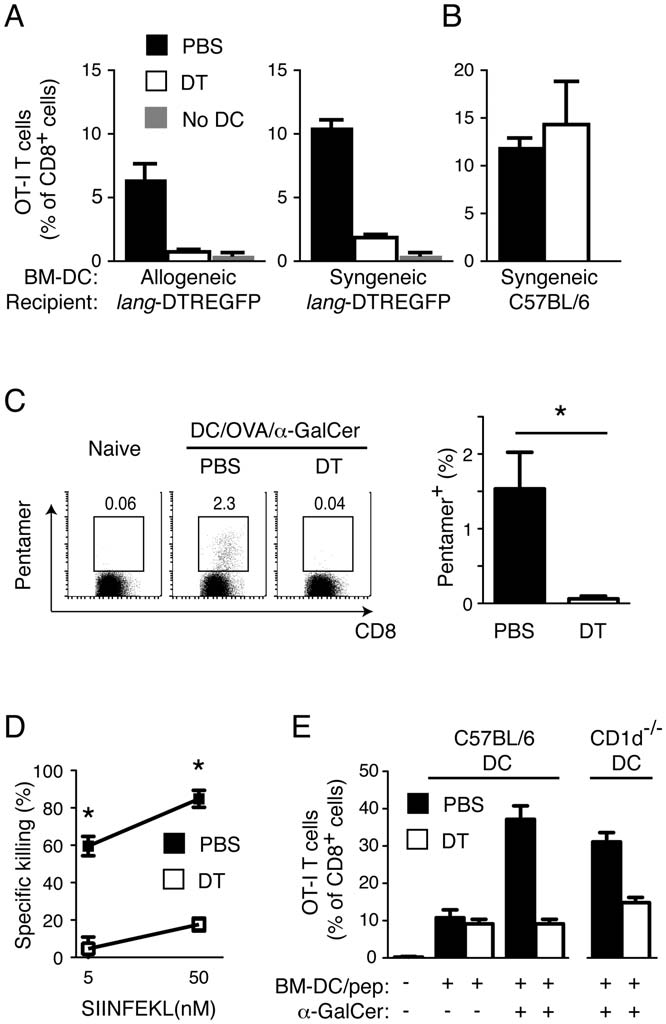
Resident langerin^+^ CD8α^+^ DCs cross-present antigen derived from injected BM-DCs to CD8^+^ T cells. (A) *Lang*-DTREGFP mice were injected *i.p.* with DT to deplete resident langerin^+^ cells, or with PBS vehicle, and then all mice were provided a cohort of transgenic OT-I T cells (CD45.1) by adoptive transfer. The following day, the mice were vaccinated *i.v.* with allogeneic (B10.Q) or syngeneic (C57BL/6j) BM-DCs loaded with OVA and α-GalCer, and the percentage of OT-I T cells determined in the blood 7 d later by flow cytometry. One representative experiment of two with 4-5 mice per group is shown. The mean percentage ± SEM is depicted. (*B*) As in *A*, except syngeneic antigen-loaded BM-DCs were injected into non-transgenic C57BL/6j mice. One representative experiment out of two with 4 mice per group is depicted with SEM. (*C*) *Lang*-DTREGFP mice were vaccinated with syngeneic BM-DCs loaded with OVA and α-GalCer as in *A* but without the transfer of transgenic OT-I T cells. The percentage of CD8^+^ T cells specific for the OVA_257–264_ peptide (SIINFEKL) was determined in the blood six days later by flow cytometry using fluorescently labeled pentamers of K^b^/OVA_257–264_. Representative dot plots from the indicated experimental groups are shown. The bar graph shows the mean percentage + SEM of pentamer positive cells. One experiment with 4 mice per group is depicted. (*D*) *In vivo* cytotoxic activity in mice from *C* was determined by measuring the relative recovery of spleen cells loaded with 50 or 5 nM of OVA_257–264_ peptide injected *i.v.* into vaccinated mice, and collected from blood 17 h later. Statistical significance was determined with a Mann-Whitney test. *p<0.05.

We next determined which population of resident APCs was responsible for capture and indirect cross-presentation of antigen derived from injected BM-DCs. Lymphoid-resident DCs expressing a homodimer of CD8α have previously been demonstrated to efficiently cross-present soluble and cell-associated antigens [5], [6], [7], [8]. In addition, we have recently shown that the ability to cross-present soluble antigen resides almost exclusively within a subpopulation of CD8α^+^ DCs that express the c-type lectin langerin (CD207) [18]. In order to determine if langerin^+^ CD8α^+^ DCs are responsible for cross-presentation of antigen derived from injected BM-DCs, we took advantage of the *lang*-DTREGFP mice [27], in which langerin-expressing DCs, including langerin^+^ CD8α^+^ DCs, can be selectively depleted by administration of *Diphtheria* toxin (DT). We treated *lang*-DTREGFP or control C57BL/6j mice with DT prior to vaccination with either syngeneic or allogeneic BM-DCs loaded with OVA and α-GalCer, and assessed priming of OT-I T cells as described above. Depletion of langerin^+^ CD8α^+^ DCs by DT injection was found to ablate OT-I T cell priming in response to allogeneic antigen-loaded BM-DCs, indicating that langerin^+^ CD8α^+^ DCs mediate indirect cross-presentation of antigen derived from the injected cells (Fig. 2*A*). Interestingly, depletion of langerin^+^ CD8α^+^ DCs also reduced priming of OT-I cells in response to syngeneic antigen-loaded BM-DCs despite these cells being capable of directly stimulating OT-I T cells (Fig. 2*A*). In contrast, administration of DT had no effect on priming of OT-I T cells in C57BL/6 mice, demonstrating that this treatment specifically affects T cell priming by depleting langerin^+^ cells (Fig. 2*B*). Priming of OVA-specific CD8^+^ T cells in the absence of transferred OT-I T cells was similarly ablated in *lang*-DTREGFP mice treated with DT and vaccinated with syngeneic BM-DCs loaded with OVA and α-GalCer (Fig. 2*C*). This correlated with a significant impairment in the ability of DT-treated mice to kill target cells loaded with OVA_257–264_-peptide (Fig. 2*D*). Transfer of antigen to resident langerin^+^ CD8α^+^ DCs is therefore a major contributor to CD8^+^ T cell stimulation in this DC vaccination strategy.

### Injected antigen-loaded DCs, and not resident DCs, prime CD4^+^ T cell responses

**Figure 3:**
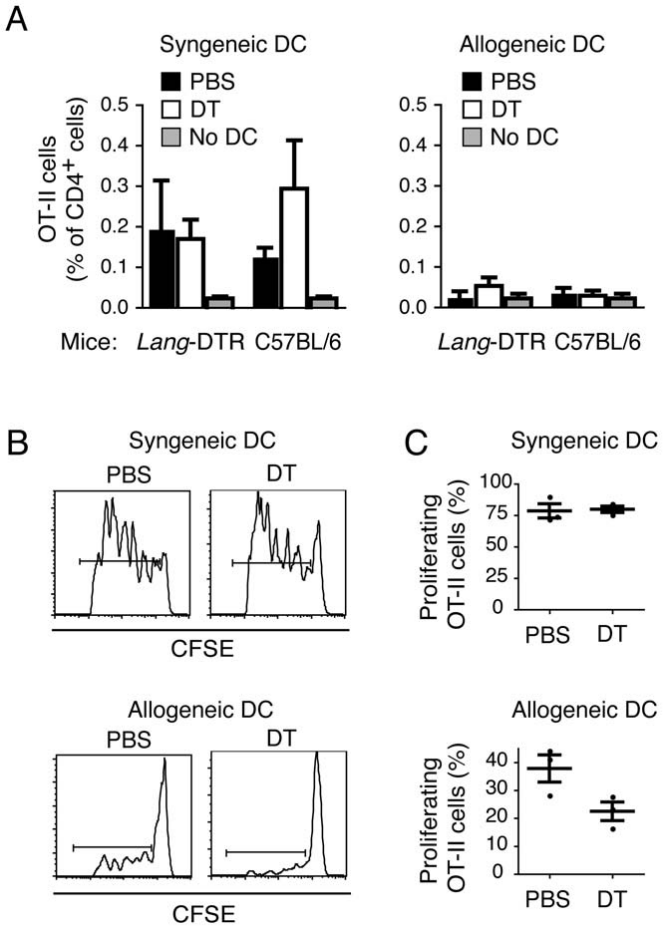
CD4^+^ T cell responses are primed by injected antigen-loaded BM-DCs with minimal involvement of resident langerin^+^ CD8α^+^ DCs. (A) *Lang*-DTREGFP mice, or C57BL/6j controls, were injected *i.p.* with DT or PBS, and were then provided a cohort of OVA-specific CD4^+^ T cells from OT-II mice. The following day, the mice were vaccinated *i.v.* with OVA- and α-GalCer-loaded BM-DCs from syngeneic (C57BL/6j), or allogeneic (B10.Q) mice, and the percentage of OT-II cells of all CD4^+^ cells determined in the blood 7 days later by flow cytometry. Mean percentages of one representative experiment of two with 5 mice per group are shown with SEM. (*B*, *C*) *Lang*-DTREGFP mice were treated with PBS or DT and then provided CFSE-labeled OT-II cells before vaccination with OVA- and α-GalCer-loaded BM-DCs from syngeneic or allogeneic mice. (*B*) Representative histogram plot showing CFSE dilution of OT-II T cells three days after administration of antigen-loaded BM-DCs. (*C*) Mean percentages of divided OT-II T cells with SEM for one representative experiment of two, with three animals in each treatment group.

To determine whether transfer of antigen is required to stimulate CD4^+^ T cell responses to injected antigen-loaded BM-DCs, and to also assess whether langerin^+^ CD8α^+^ DCs are involved, we examined priming of a cohort of OVA-specific transgenic CD4^+^ T cells (OT-II cells) in C57BL/6j mice and *lang*-DTREGFP mice. Injection of syngeneic BM-DCs loaded with OVA and α-GalCer resulted in priming of OT-II cells in C57BL/6j and untreated *lang*-DTREGFP recipients, with enhanced accumulation of OT-II cells in the blood at day 7 compared to non-vaccinated mice (Fig. 3*A*). However, this response was not dependent on resident langerin^+^ CD8α^+^ DCs because prior depletion of langerin^+^ cells in *lang*-DTREGFP mice had no effect on the induced response (Fig. 3*A*). In addition, OT-II T cell priming was only induced when direct stimulation by the injected DCs was possible, as no expansion of the OT-II T cell population was observed in response to antigen-loaded allogeneic BM-DCs (Fig. 3*A*). Because the overall OT-II T cell response was low in these experiments, we performed similar experiments in mice receiving CFSE-labeled OT-II T cells, and used dilution of the CFSE dye as a measure of cell division to assess OT-II T cell activation. Using this more sensitive method, we confirmed the previous finding that maximal OT-II T cell priming occurred when the injected BM-DCs were capable of stimulating OT-II T cells directly (Fig. 3*B*), although there was some limited T cell proliferation in response to allogeneic BM-DC which may be attributed to antigen transfer (Fig. 3*B*). This latter response was partially ablated upon depletion of langerin^+^ cells (Fig. 3*B and C*), although the difference between DT-treated and not-treated mice did not reach significance (Fig. 3*C*). In contrast, the larger response induced to syngeneic BM-DCs was not obviously sensitive to langerin depletion (Fig. 3*B and C*). Overall, injected BM-DCs are primarily responsible for directly stimulating CD4^+^ T cells, whereas a major proportion of the CD8^+^ T cell response is stimulated as a consequence of antigen transfer from injected cells to resident langerin^+^ CD8α^+^ DCs.

### Resident DCs need a maturation signal to cross-present antigen acquired from injected BM-DCs

**Figure 4:**
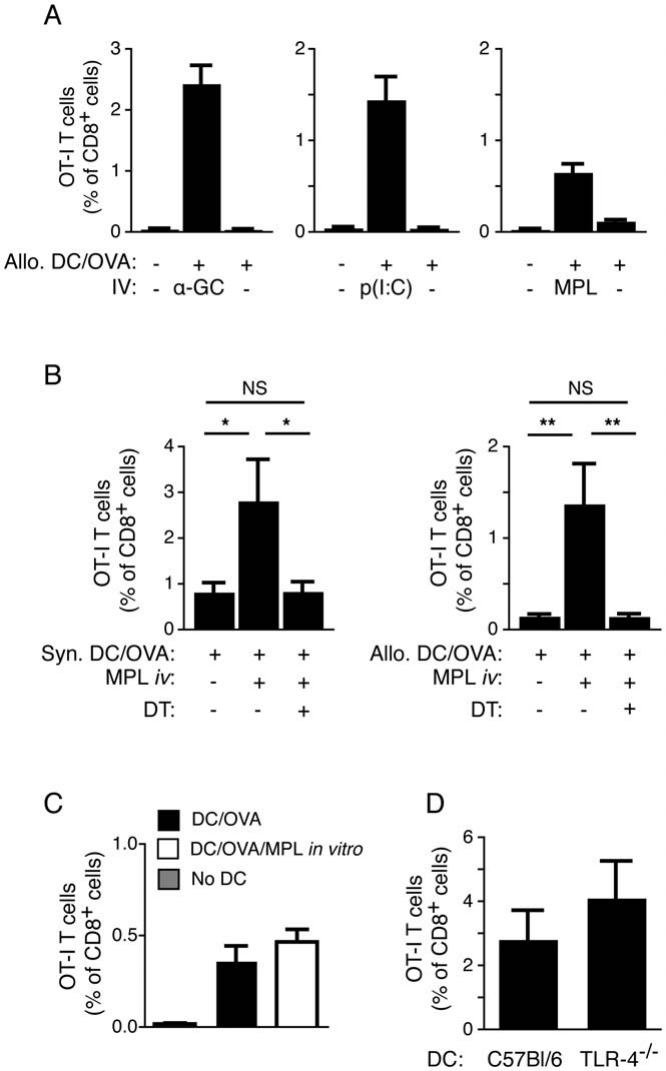
Resident langerin^+^ DCs need a maturation signal to cross-present antigen derived from antigen-loaded BM-DCs. (*A*) C57BL/6j mice were provided a cohort of OT-I T cells and vaccinated *i.v.* with allogeneic (B10.Q) BM-DCs with or without OVA. The mice were injected in the contralateral tail vein with either α-GalCer, p(I:C), or MPL and the percentage of OT-I T cells of all CD8^+^ cells was determined in the blood 7 d later. One representative experiment out of two with 4–5 mice per group is shown with SEM. (*B*) *Lang*-DTREGFP mice were treated with DT, or PBS vehicle, provided a cohort of OT-I T cells, and then administered OVA-loaded BM-DCs from syngeneic or allogeneic mice with or without injection of MPL in the contralateral vein. The percentage of OT-I T cells of all CD8^+^ cells was determined in the blood 7 d later. Pooled data from two experiments with 4–5 mice per group is depicted with SEM. Statistical significance was determined with a Kruskal-Wallis test and Dunn's post test. *p<0.05, **p<0.01, NS =  not significant. (*C*) C57BL/6j mice received a cohort of OT-I T cells as above, and were vaccinated with syngeneic OVA-loaded BM-DCs that had been incubated with 100 ng/ml of MPL for 16 h prior to injection, or left untreated. The mean percentage of OT-I T cells of all CD8^+^ cells in the blood 7 d later is depicted using pooled data from three experiments with 4–5 mice per group, with SEM. (*D*) C57BL/6j mice received OT-I T cells as above and were vaccinated with OVA- and α-GalCer-loaded BM-DCs derived from C57BL/6j or TLR-4^−/−^ mice. The mean percentage of OT-I T cells of all CD8^+^ cells in the blood 7 d later is depicted with SEM from one representative experiment out of two.

DC maturation is a requirement for optimal stimulation of naïve T cells. It is therefore likely that resident DCs are in similar need of a maturation signal in order to stimulate naïve T cells with antigen acquired from injected BM-DCs. To address whether this was the case, we assessed indirect cross-priming of OT-I T cells in C57BL/6j mice following injection of allogeneic OVA-loaded BM-DCs in the presence or absence of different adjuvant compounds known to mature DC. These compounds were injected *i.v.* at the same time as the BM-DCs in order to provide systemic access to the lymphoid tissues, but injections were into the contralateral tail vein to limit direct interactions between the compounds and the injected cells. Injection of allogeneic OVA-loaded BM-DCs in the presence of the adjuvant α-GalCer induced significant OT-I T cell priming. However, this response was almost undetectable in the absence of α-GalCer (or other maturation signals) (Fig. 4*A*), suggesting that resident DCs require an *i*NKT cell-mediated maturation signal in order to engage in T cell priming. It was possible that even a limited amount of binding of α-GalCer to the injected cells *in vivo* could make the injected cells susceptible to *i*NKT cell-mediated lysis, thereby facilitating the release of antigen available to resident DCs. In order to rule out this potential contribution to the induced response, we repeated the above experiment using *i.v.* injection of the TLR ligands p(I:C) or MPL as adjuvant compounds, thereby avoiding the involvement of *i*NKT cells altogether. Intravenous administration of each of these TLR ligands also enhanced the ability of the resident DCs to indirectly cross-present antigen derived from injected allogeneic BM-DCs (Fig. 4*A*). The enhancement mediated by MPL was completely negated by prior treatment with DT in *lang*-DTREGFP recipients, regardless of whether syngeneic or allogeneic BM-DCs were injected (Fig. 4*B*), indicating that MPL enhance CD8^+^ T cell priming by maturing the langerin^+^ CD8α^+^ DCs. It is unlikely that the *i.v.* administered MPL contributed to the T cell response by directly stimulating the injected BM-DCs, as treatment of the OVA-loaded BM-DCs with MPL *in vitro* before injection failed to significantly improve the OT-I response (Fig. 4*C*). Also, responses to OVA-loaded BM-DCs from TLR4-deficient mice, which cannot respond directly to MPL, could be enhanced by *i.v.* administration of MPL to levels similar to BM-DCs from TLR4-sufficient mice (Fig. 4*D*). Overall, these data suggest that resident langerin^+^ CD8α^+^ DCs require a maturation signal to participate in indirect cross-presentation.

### Resident antigen-presenting cells present α-GalCer acquired from injected α-GalCer-loaded BM-DCs

**Figure 5:**
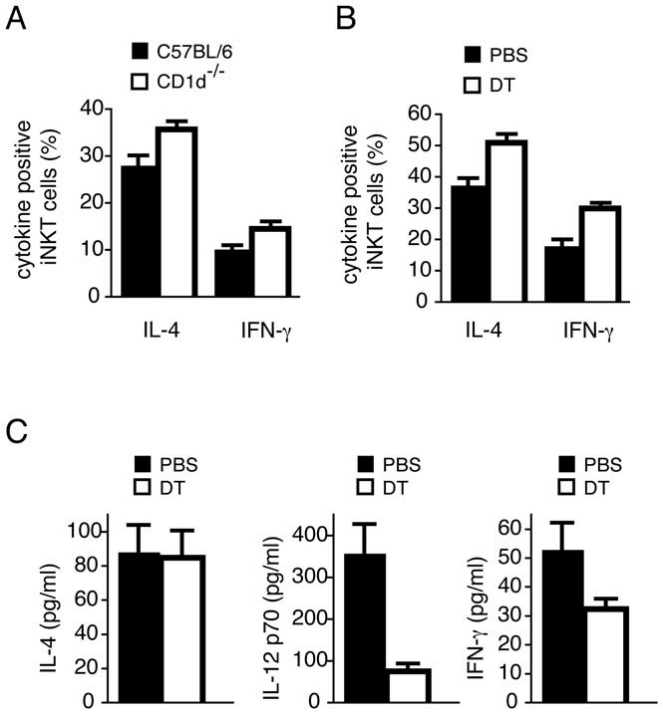
Resident APCs acquire α-GalCer from α-GalCer-loaded BM-DCs in vivo. (*A*) BM-DCs derived from C57BL/6j (black bars) or CD1d^−/−^ (white bars) mice were loaded with 200 ng/ml of α-GalCer for 16 h and injected *i.v.* into C57BL/6j mice. The percentage of splenic *i*NKT cells expressing intracellular IL-4 or IFN-γ was determined 2 h later by flow cytometry. One representative experiment out of two with three mice per group is depicted, with SEM. (*B*) CD1d^−/−^ BM-DCs were incubated with α-GalCer as above and injected into *lang*-DTREGFP mice, which had been injected with either PBS (black bars) or DT (white bars). The percentage of *i*NKT cells expressing intracellular IL-4 or IFN-γ was determined 2 h later by flow cytometry. One representative experiment out of two with three mice per group is depicted with SEM. (*C*) *Lang*-DTREGFP mice were treated with PBS (black bars) or DT (white bars) and then injected *i.v.* with α-GalCer-loaded BM-DCs from C57BL/6j mice. Serum concentrations of IL-4, IL-12 p70, and IFN-γ were determined 2 h, 5 h, and 10 h later respectively. One representative experiment out of three with 3-5 mice per group is depicted with SEM.

We next examined how loading α-GalCer onto the injected BM-DCs serves to enhance the stimulatory capacity of resident CD8^+^ langerin^+^ DC. The adjuvant effect of α-GalCer-mediated *i*NKT cell activation requires that α-GalCer is presented on CD1d molecules by APCs engaged in T cell stimulation [20], [22], [31]. This stimulates *i*NKT cells to become activated and secrete IL-4 and IFN-γ, and the activated *i*NKT cells in turn stimulate enhanced APC function, including secretion of proinflammatory cytokines such as IL-12 [32], [33], [34], [35], [36]. The release of IL-12 then instructs NK cells to produce more IFN-γ [37]. While release of cytokines into the environment is sufficient to induce up-regulation of costimulatory molecules such as CD80 and CD86 on local APCs, the ability of these cells to promote T cell responses is critically dependent on direct cellular interactions between APC and *i*NKT cells, with CD40/CD40L interactions particularly crucial [20], [22], [38]. We therefore hypothesized that resident DCs must acquire α-GalCer from injected DCs in order to interact with *i*NKT cells directly. To test this hypothesis, we examined whether injection of α-GalCer-loaded CD1d-deficient (CD1d^−/−^) BM-DCs, which cannot directly present α-GalCer to *i*NKT cells, can activate *i*NKT cells in C57BL/6j recipients. Similar percentages of cytokine-positive *i*NKT cells were induced by α-GalCer-loaded CD1d^−/−^ and wild-type BM-DCs, when intracellular antibody staining for IFN-γ and IL-4 was used as a measure for *i*NKT cell activity (Fig. 5*A*). These data suggest that α-GalCer is transferred from the injected BM-DC to resident CD1d^+^ APCs that can engage directly in stimulating *i*NKT cells. We next examined the role of langerin^+^ CD8α^+^ DCs in this process by injecting α-GalCer-loaded CD1d^−/−^ BM-DCs into DT-treated or untreated *lang*-DTREGFP mice. Similar percentages of activated *i*NKT cells (as measured by intracellular cytokine staining) were seen regardless of DT treatment, indicating that langerin^+^ CD8α^+^ DCs were dispensable for initial *i*NKT cell activation (Fig. 5*B*). Accordingly, serum levels of IL-4 two hours after administration of BM-DCs were similar in depleted and non-depleted mice (Fig. 5*C*). However, depletion of langerin^+^ cells severely reduced the level of IL-12p70 released into the serum (Fig. 5*C*), demonstrating an important role of langerin^+^ CD8α^+^ DCs in the production of this cytokine in response to α-GalCer as we have recently reported [18]. Serum levels of IFN-γ were also reduced in recipients depleted of langerin^+^ cells, which is likely to reflect reduced trans-activation of NK cells as a consequence of lower IL-12 levels (Fig. 5*C*). Overall, these data demonstrate that α-GalCer is transferred from injected BM-DCs to APC populations within the lymphoid tissue that are capable of stimulating cytokine production by *i*NKT cells. Crucially, the subsequent release of IL-12p70, which may help in driving cross-primed responses [39], is largely dependent on the presence of langerin^+^ CD8α^+^ DCs among the recipient APCs.

### Resident langerin^+^ CD8α^+^ DCs can acquire cellular material from injected BM-DCs

**Figure 6:**
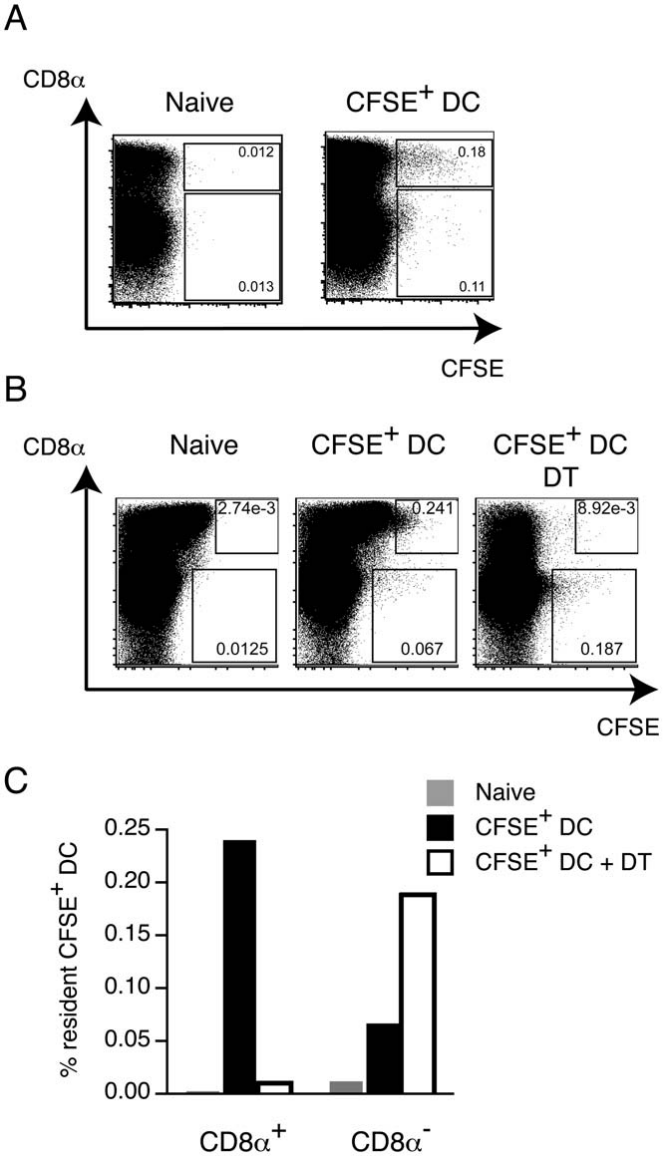
Injected BM-DCs are taken up by resident DCs. (A) Pools (n = 2) of C57BL/6j mice (CD45.2) were injected with CFSE-labeled BM-DCs from CD45.1 congenic mice, or left untreated. Spleens were removed 16 h later and CD11c^+^ cells were isolated using magnetic antibody-coated beads. Depicted dot plots show CFSE fluorescence in resident (CD45.2^+^) CD11c^+^ cells. One representative experiment of two is depicted. (*B*) CFSE-labeled BM-DCs from CD45.1 congenic mice were injected into *lang*-EGFPDTR mice with or without prior depletion of langerin^+^ cells by DT, and the acquisition of CFSE by resident DCs determined as above. (*C*) Graphical representation of CFSE acquisition by resident CD8α^+^ and CD8α^−^ DCs in *lang*-DTREGFP mice with or without prior depletion of langerin^+^ cells.

The antigen-loaded BM-DCs used in all of the previous experiments were extensively washed before administration. Injection of the final washes, or supernatants from cells subsequently cultured in vitro, was not capable of inducing proliferation of OT-I cells in vivo, ruling out the possibility that OVA was ‘leaching’ as free antigen from the cells over time (data not shown). Some α-GalCer did accumulate in culture supernatants, but at levels that were insufficient to account for the *in vivo* activities we have observed (data not shown). We therefore investigated the possibility that the antigens were transferred in an exchange of cellular material between injected and resident cells. To determine whether any cellular material could be exchanged in this manner, BM-DCs derived from CD45.1 congenic mice were CFSE-labeled and injected into CD45.2^+^ C57BL/6j mice, and the acquisition of CFSE fluorescence by resident (CD45.2^+^) DCs determined 16 h later. Analysis of CD11c^+^ cells in the spleens of recipient animals showed that CFSE fluorescence was acquired by resident CD8α^+^ DCs as well as CD8α^−^ DCs (Fig. 6A), with fluorescence intensity highest in CD8α^+^ cells. To establish if CFSE fluorescence was acquired by the langerin^+^ subset, we similarly injected CFSE-labeled CD45.1 BM-DCs into *lang*-EGFPDTR mice. In these mice, all langerin^+^ CD8α^+^ resident DCs are weakly GFP positive (Fig. 6B, *naïve*). As above, a subset of resident CD8α^+^ DCs acquire CFSE fluorescence upon injection of CFSE-labeled BM-DCs (Fig. 6B, *CFSE^+^ DC*). However, the acquisition of CFSE fluorescence by resident CD8α^+^ DCs is ablated upon prior depletion of langerin^+^ cells by DT (Fig. 6B, *CFSE^+^ DC + DT*, and Fig. 6C), suggesting cellular material is mainly acquired by CD8α^+^ DCs expressing langerin.

## Discussion

In this report we investigated the contribution of endogenous lymphoid-resident DCs to T cell priming after vaccination with antigen-loaded BM-DCs. We demonstrate that CD8^+^ T cells primed in response to *i.v.* injection of antigen-loaded BM-DCs can be primed by a subset of resident DCs expressing CD8α and langerin. However, this subset of DCs only contributes to T cell priming if a potent maturation signal is received in combination with the administered BM-DCs. We also demonstrate that antigen transfer is not simply limited to protein antigens, as the glycolipid α-GalCer is also acquired and presented by endogenous DC, which facilitates licensing of the resident DCs as a consequence of engaging *i*NKT cells.

Two models were used to demonstrate that resident DCs acquire antigen from injected BM-DCs. The first model involved vaccination with allogeneic OVA-loaded BM-DCs, which were unable to directly stimulate OVA-specific OT-I T cells, and therefore required antigen to be transferred to host APCs to induce expansion of the transgenic T cell population. While this model showed that OT-I cells could indeed be primed if an adjuvant was provided, it is possible that this response was inflated because the allogeneic BM-DCs were lysed by NK cells, promoting release of antigen to the resident DCs [6]. In order to avoid this possibility, and characterize the resident DC population responsible for CD8^+^ T cell priming in more detail, we used a second model in which the recipient DCs were selectively depleted. Our previous studies have shown that langerin^+^ CD8α^+^ DCs in the spleen are critically involved in cross-presentation of systemic antigens and IL-12 induction [18]. We therefore examined DC vaccination in *lang*-DTREGFP mice in which all DCs expressing langerin could be depleted by injection of DT [27]. Typically, approximately 90% of the splenic langerin^+^ CD8α^+^ DC subset is depleted for at least two days in response to DT (Fig. S1). In contrast, DT injection does not affect langerin^−^ CD8α^+^ DCs, CD8α^−^ DCs, or the injected DCs themselves [18]. In addition to being expressed by a subset of CD8α^+^ DCs in the spleen, langerin is expressed by Langerhans cells in the epidermis, and a subset of dermal and lung DCs additionally expressing CD103 [27], [40], [41], [42], [43]. These DC subsets may therefore also be depleted by DT administration, and may be of relevance to the response to injected BM-DCs. BM-DCs injected *i.v.* do initially accumulate in much higher numbers in the lungs compared to the spleen, but then disappear from the lungs within the first 24 h. In contrast, the number of injected BM-DCs in the spleen remains high for several days, and the spleen contains the largest number of injected BM-DCs a day after injection [44]. Although T cells are likely to be primed in the lung-draining lymph nodes as well as in the spleen following *i.v.* administration of antigen-loaded BM-DCs, the size of the spleen relative to lymph nodes combined with the continuous presence of the injected DCs, make the spleen the primary site of T cell priming [44]. Using the *lang*-DTREGFP model, depletion of langerin^+^ DCs before *i.v.* injection of OVA and α-GalCer-loaded allogeneic BM-DCs completely ablated OT-I T cell priming, and reduced OT-I T cell priming more than 75% when syngeneic BM-DCs were used (Fig. 2A). Resident langerin^+^ DCs are therefore responsible for priming the majority of the OVA-specific CD8^+^ T cell response even when syngeneic BM-DCs were used, and NK-mediated killing of the injected BM-DCs is not required for antigen transfer.

Lymphoid resident DCs did not prime CD8^+^ T cells after injection of antigen-loaded BM-DCs unless the injected DCs are administered with a TLR agonist or α-GalCer. As illustrated using MPL, the adjuvant effect of co-administered TLR ligand was negated by prior depletion of langerin^+^ DCs, suggesting that MPL enhances CD8^+^ T cell priming by inducing maturation of resident langerin^+^ DCs, and not by the stimulation of other cell types, or the generation of an inflammatory environment. Nor is the effect of MPL due to maturation of the injected BM-DCs because responses to TLR-4 deficient BM-DCs were similarly enhanced when MPL was co-administered. Surprisingly, maturation of BM-DCs prior to injection had a very limited effect on OT-I T cell priming despite upregulation of costimulatory markers on the cells before injection, and induction of release of cytokines such as IL-12 and IL-6 in vitro (not shown). In fact, our preliminary data suggest that injected BM-DCs that reach the spleen display a mature phenotype irrespective of whether they were matured in vitro prior to injection (data not shown). In contrast to OT-I T cells, only very few OT-II T cells were primed by allogeneic BM-DCs loaded with OVA- and α-GalCer, and this response was only mildly impacted by depletion of langerin^+^ DCs. However, OT-II T cells were efficiently primed by syngeneic BM-DCs loaded with OVA and α-GalCer, suggesting that it is the injected BM-DCs, and not lymphoid-resident DCs, that predominate in the stimulation of naïve CD4^+^ T cells.

The mechanism of antigen transfer in our experiments remains unclear. It has been shown that membrane fragments can be exchanged between live DCs following direct cell to cell contact [45], which may result in transfer of antigens presented on the surface. Similarly, antigens can be incorporated into exosomes released from a variety of hematopoietic and non-hematopoietic cell types (including DCs), and taken up by APCs [46]. Antigen can also be transferred by the uptake of DCs undergoing apoptosis, or as apoptotic bodies [14], [47]. Our results showed that splenic APCs acquired fluorescence from injected CFSE-labeled BM-DCs, suggesting uptake of cellular material. Our experiments also showed that resident langerin^+^ CD8α^+^ DCs acquired the most fluorescence from the CFSE-labeled BM-DCs; some CD8α^−^ DCs acquired fluorescence, but the intensity was lower, suggesting either less efficient uptake, a qualitatively different mechanism of uptake, or efficient uptake and subsequent degradation.

It is well-described that the *in vivo* priming of CD8^+^ T cells in response to intravenous injection of soluble or cell-associated antigens is critically dependent on CD8α^+^ DCs with capacity for cross-presentation [5], [6], [7], [8]. On the other hand, priming of CD4^+^ T cells in response to the same antigen sources is minimally affected by prior depletion of CD8α^+^ DCs, suggesting that other, CD8α-negative, APCs engage more efficiently in MHC class II presentation *in vivo* [18], [48]. Inefficient targeting of antigen to APCs with high propensity for CD4^+^ T cell priming could therefore provide one explanation for the limited CD4^+^ T cell response primed as a consequences of antigen transfer in our model. It is possible that these differences in APC function can be explained not just by inherent differences in processing and presentation capacity, but also by differences in location in the lymphoid tissue, and capacity to respond to maturation stimuli. Thus, while it is clear that CD8α^+^ DC possess superior cross-presenting function, the splenic langerin^+^ fraction of this subset are also positioned within the marginal zone [49], and are therefore well-positioned to acquire antigens from the circulation to divert into the MHC class I presentation pathway. In fact, langerin^+^ CD103^+^ CD8α^+^ DCs within the marginal zone have been shown to be particularly efficient at acquiring cellular material [50], which may be of particular relevance in our DC vaccination model. The CD8α^+^ DC population has also been shown to produce CCL17 in response to *i*NKT cell activation, which favors attraction of naïve CD8^+^ T cells via CCR4 [31], and the langerin^+^ subset responds more quickly to *i*NKT cell-mediated maturation stimuli in terms of costimulatory marker upregulation, and CD40 expression, than other DCs of the spleen [18]. Collectively these factors may favor induction of CD8^+^ T cell responses by langerin^+^ CD8α^+^ DCs, rather than CD4^+^ T cell responses, although our data show that some minimal CD4^+^ T cell stimulation attributed to langerin^+^ CD8α^+^ DCs may also occur.

Our findings do contrast with previous reports demonstrating MHC class II presentation as a consequence of antigen transfer from injected BM-DCs to resident DCs [14], [15]. The reason for this discrepancy is not known, but may reflect the different forms of antigen and concentration used, particularly the use of high concentrations of an MHC class II-binding peptide (MCC_88–103_) in one study where priming of CD4^+^ T cells by endogenous DCs was actually observed [15]. Alternatively, it has also been shown that OT-II T cells display low sensitivity to low doses of antigen when compared to OT-I T cells in vivo [51], which is supported by our studies (Fig. 3, and [18]), and may explain the negligible CD4^+^ T cell response induced as a consequence of antigen transfer between DCs in our experimental system. Further experimentation is required to resolve this issue.

Interestingly, vaccination with α-GalCer-loaded CD1d^−/−^ BM-DCs induced *i*NKT cell activation to similar levels as α-GalCer-loaded wild-type BM-DCs. As presentation of α-GalCer to *i*NKT cells is dependent on CD1d, this finding can only be explained by transfer of α-GalCer from the injected CD1d-negative BM-DCs to CD1d-positive resident APCs. Resident DCs may acquire α-GalCer following phagocytosis of whole antigen-loaded BM-DCs as previously suggested for the transfer of α-GalCer from loaded tumor cells to resident DCs [52]. However, despite the proposed superior ability of CD8α^+^ DCs to phagocytose dying cells [6], [53], which is supported by our observations (Fig. 6), the activation of *i*NKT cells *per se* is not dependent on the langerin^+^ fraction of this population, as the depletion of these cells in *lang*-DTREGFP mice did not influence initial *i*NKT cell priming. We cannot rule out the possibility that other APC subsets such as CD11b^+^ CD4^+^ splenic DCs or marginal zone macrophages, can take up cellular material from antigen-loaded BM-DCs for presentation of α-GalCer. In this context, subcapsular sinus CD169^+^ macrophages were recently shown to mediate early activation of *i*NKT cells in lymph nodes in response to particulate α-GalCer [54].

Although not important for initial *i*NKT cell activation, α-GalCer-mediated IL-12 production was critically dependent on resident langerin^+^ CD8α^+^ DC, as IL-12 production was severely reduced in DT treated animals. This is consistent with our previous studies showing that the absence of langerin^+^ cells has no impact on the ability of injected free α-GalCer to elicit *i*NKT cells to release IL-4 and IFN-γ, but that the subsequent release of large quantities of IL-12p70 into the serum, which is mediated through interaction of activated *i*NKT cells with APCs, is severely impaired [18]. We also noted in these earlier studies a heightened propensity of langerin^+^ CD8α^+^ DC to upregulate CD40 in response to free α-GalCer, which potentially explains the significant role these cells play in driving IL-12p70 release. The failure to produce IL-12p70 also prevented the release of large quantities of IFN-γ into the serum, which has been previously described to be secreted by NK cells [37].

Using conditional ablation of langerin^+^ cells in *lang*-DTREGFP mice, we find that a significant proportion of the CD8^+^ T cells responding to intravenous injection of antigen-loaded BM-DCs are primed by endogenous langerin^+^ DCs. Yet, this subset of DCs only contributes to T cell priming if a potent maturation signal is received in combination with the administered BM-DCs. As targeting of antigen to immature DCs *in vivo* has been shown to induce T cell tolerance [55], [56], [57], our results underscore the utility of providing a stimulus for endogenous resident DCs in DC vaccination protocols.

## Supporting Information

Figure S1 **Splenic CD8^+^ langerin^+^ DCs are efficiently depleted by diphtheria toxin injections.** F1 crosses of *Lang*-DTREGFP x *Lang*-EGFP were injected *i.p.* with 350 ng of DT 48 h and 24 h prior to analysis. Single cell suspensions of spleen cells were generated by Liberase and DNAse treatment as described in Materials and Methods. Cells were stained with antibodies against CD11c and CD8 as well as PI for live/dead cell exclusion. (*A*) Representative dot plots showing the percentage of CD11c high cells positive for CD8 and langerin-eGFP in DT treated or non-treated mice. (*B*) Percentage of CD8^+^ langerin^+^ cells of CD11c high cells in individual mice from a total of three experiments with three mice per group.
(TIF)
