# Supplementary figures and images for: Exploiting the Role of Endogenous Lymphoid-Resident Dendritic Cells in the Priming of NKT Cells and CD8+ T Cells to Dendritic Cell-Based Vaccines
Source: PLoS One. 2011 Mar 31;6(3):e17657. doi: 10.1371/journal.pone.0017657 (PMC3069042; doi:10.1371/journal.pone.0017657)

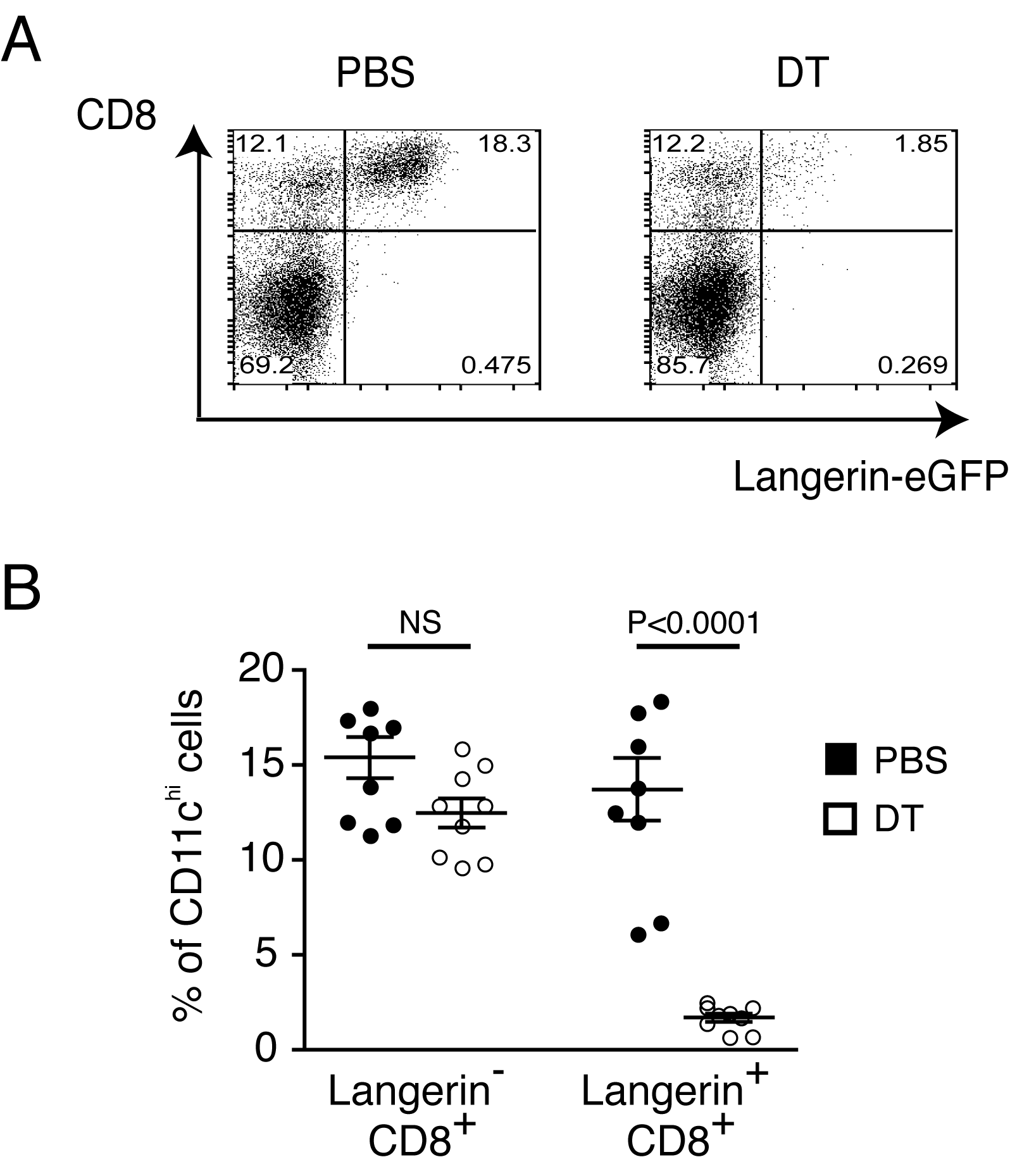

Supplement: Figure S1 — Splenic CD8+ langerin+ DCs are efficiently depleted by diphtheria toxin injections. F1 crosses of Lang-DTREGFP x Lang-EGFP were injected i.p. with 350 ng of DT 48 h and 24 h prior to analysis. Single cell suspensions of spleen cells were generated by Liberase and DNAse treatment as described in Materials and Methods. Cells were stained with antibodies against CD11c and CD8 as well as PI for live/dead cell exclusion. (A) Representative dot plots showing the percentage of CD11c high cells positive for CD8 and langerin-eGFP in DT treated or non-treated mice. (B) Percentage of CD8+ langerin+ cells of CD11c high cells in individual mice from a total of three experiments with three mice per group. (TIF) [file pone.0017657.s001.tif]
